# Magnetically encoded 3D mesostructure with high-order shape morphing and high-frequency actuation

**DOI:** 10.1093/nsr/nwac163

**Published:** 2022-08-16

**Authors:** Rui Li, Cong Zhang, Jiawen Li, Yachao Zhang, Shunli Liu, Yanlei Hu, Shaojun Jiang, Chao Chen, Chen Xin, Yuan Tao, Bin Dong, Dong Wu, Jiaru Chu

**Affiliations:** Hefei National Laboratory for Physical Sciences at the Microscale, CAS Key Laboratory of Mechanical Behavior and Design of Materials, Key Laboratory of Precision Scientific Instrumentation of Anhui Higher Education Institutes, Department of Precision Machinery and Precision Instrumentation, University of Science and Technology of China, Hefei 230027, China; Hefei National Laboratory for Physical Sciences at the Microscale, CAS Key Laboratory of Mechanical Behavior and Design of Materials, Key Laboratory of Precision Scientific Instrumentation of Anhui Higher Education Institutes, Department of Precision Machinery and Precision Instrumentation, University of Science and Technology of China, Hefei 230027, China; Hefei National Laboratory for Physical Sciences at the Microscale, CAS Key Laboratory of Mechanical Behavior and Design of Materials, Key Laboratory of Precision Scientific Instrumentation of Anhui Higher Education Institutes, Department of Precision Machinery and Precision Instrumentation, University of Science and Technology of China, Hefei 230027, China; Hefei National Laboratory for Physical Sciences at the Microscale, CAS Key Laboratory of Mechanical Behavior and Design of Materials, Key Laboratory of Precision Scientific Instrumentation of Anhui Higher Education Institutes, Department of Precision Machinery and Precision Instrumentation, University of Science and Technology of China, Hefei 230027, China; Hefei National Laboratory for Physical Sciences at the Microscale, CAS Key Laboratory of Mechanical Behavior and Design of Materials, Key Laboratory of Precision Scientific Instrumentation of Anhui Higher Education Institutes, Department of Precision Machinery and Precision Instrumentation, University of Science and Technology of China, Hefei 230027, China; Hefei National Laboratory for Physical Sciences at the Microscale, CAS Key Laboratory of Mechanical Behavior and Design of Materials, Key Laboratory of Precision Scientific Instrumentation of Anhui Higher Education Institutes, Department of Precision Machinery and Precision Instrumentation, University of Science and Technology of China, Hefei 230027, China; Hefei National Laboratory for Physical Sciences at the Microscale, CAS Key Laboratory of Mechanical Behavior and Design of Materials, Key Laboratory of Precision Scientific Instrumentation of Anhui Higher Education Institutes, Department of Precision Machinery and Precision Instrumentation, University of Science and Technology of China, Hefei 230027, China; Hefei National Laboratory for Physical Sciences at the Microscale, CAS Key Laboratory of Mechanical Behavior and Design of Materials, Key Laboratory of Precision Scientific Instrumentation of Anhui Higher Education Institutes, Department of Precision Machinery and Precision Instrumentation, University of Science and Technology of China, Hefei 230027, China; Hefei National Laboratory for Physical Sciences at the Microscale, CAS Key Laboratory of Mechanical Behavior and Design of Materials, Key Laboratory of Precision Scientific Instrumentation of Anhui Higher Education Institutes, Department of Precision Machinery and Precision Instrumentation, University of Science and Technology of China, Hefei 230027, China; Hefei National Laboratory for Physical Sciences at the Microscale, CAS Key Laboratory of Mechanical Behavior and Design of Materials, Key Laboratory of Precision Scientific Instrumentation of Anhui Higher Education Institutes, Department of Precision Machinery and Precision Instrumentation, University of Science and Technology of China, Hefei 230027, China; Hefei National Laboratory for Physical Sciences at the Microscale, CAS Key Laboratory of Mechanical Behavior and Design of Materials, Key Laboratory of Precision Scientific Instrumentation of Anhui Higher Education Institutes, Department of Precision Machinery and Precision Instrumentation, University of Science and Technology of China, Hefei 230027, China; Hefei National Laboratory for Physical Sciences at the Microscale, CAS Key Laboratory of Mechanical Behavior and Design of Materials, Key Laboratory of Precision Scientific Instrumentation of Anhui Higher Education Institutes, Department of Precision Machinery and Precision Instrumentation, University of Science and Technology of China, Hefei 230027, China; Hefei National Laboratory for Physical Sciences at the Microscale, CAS Key Laboratory of Mechanical Behavior and Design of Materials, Key Laboratory of Precision Scientific Instrumentation of Anhui Higher Education Institutes, Department of Precision Machinery and Precision Instrumentation, University of Science and Technology of China, Hefei 230027, China

**Keywords:** morphable 3D mesostructures, magnetically encoded, high-order deformation, high-frequency actuation

## Abstract

Inspired by origami/kirigami, three-dimensional (3D) mesostructures assembled via a mechanics-guided approach, with reversible and maneuverable shape-morphing capabilities, have attracted great interest with regard to a broad range of applications. Despite intensive studies, the development of morphable 3D mesostructures with high-order (multi-degree-of-freedom) deformation and untethered high-frequency actuation remains challenging. This work introduces a scheme for a magnetically encoded transferable 3D mesostructure, with polyethylene terephthalate (PET) film as the skeleton and discrete magnetic domains as actuation units, to address this challenge. The high-order deformation, including hierarchical, multidirectional and blending shape morphing, is realized by encoding 3D discrete magnetization profiles on the architecture through ultraviolet curing. Reconfigurable 3D mesostructures with a modest structural modulus (∼3 GPa) enable both high-frequency (∼55 Hz) and large-deformation (∼66.8%) actuation under an alternating magnetic field. Additionally, combined with the shape-retention and adhesion property of PET, these 3D mesostructures can be readily transferred and attached to many solid substrates. On this basis, diverse functional devices, including a switchable colour letter display, liquid mixer, sequential flashlight and biomimetic sliding robot, are demonstrated to offer new perspectives for robotics and microelectronics.

## Teaser

This work presents a 3D morphable mesostructure capable of high-order shape morphing and high-frequency actuation with large deformation, which is applied for multiple functional devices.

## INTRODUCTION

Smart actuators, capable of transforming external stimuli (e.g. light [1,2], temperature [3,4], pH [5,6] and magnetic field [7,8]) into mechanical deformation, have found broad application in artificial muscles [9], robotics [10] and biomedicine [11]. Among the diverse strategies for forming actuators, three-dimensional (3D) functional mesostructures [12–20] assembled from 2D precursors through compressive force are compelling owing to their compatibility with most advanced planar semiconductor technologies and functional materials [12]. To endow 3D mesostructures with reconfigurability and multifunctionality, strategies relying on stimuli-responsive materials, substrate design and piezoelectric materials have been intensively studied. As common stimuli-responsive materials, shape memory polymers (SMPs) [21] and liquid crystal elastomers (LCEs) [18,22] are generally used to create 3D morphable mesostructures, but the morphing speed is slow due to the time-consuming heat-conduction process. By sequentially releasing the pre-stretched substrate [13] or predesigned substrate [23], the same 2D precursor can morph into various configurations, but is still subject to deformation speed because of manual operation. Although the morphable 3D architectures made by piezoelectric material [14,15] can produce switching and displacement responses rapidly, they are restricted to wired actuation and small deformation, which will encounter problems when working in confined and enclosed spaces. Besides, the above-mentioned strategies suffer from having few configurations for a low degree of freedom (DOF) of deformation. Therefore, these design principles are trade-offs among reconfiguration speed, multiple configurations and wired control, which restrict the 3D mesostructures to simple applications.

In comparison with the above methods, magnetic actuation is more suitable for 3D morphable mesostructures due to its precise, fast and wireless actuation [24]. Recently, by programming magnetic liquid crystal molecules into complex 2D patterns, Li *et al*. [18,22] developed a kind of 3D mesostructure, induced by compressive forces, that can be transformed into multiple configurations in response to magnetic force. Zhang *et al*. [25] realized shape-morphing magnetic 3D mesostructures by plating a layer of ferromagnetic material on the structures, thereby switching between several configurations under a gradient magnetic field. Although these approaches enable wireless control and multiconfiguration, a homogeneous distribution of magnetic moments assigned to the structures severely restricts the degree of reconfiguration freedom and further high-order shape morphing. In addition, to control magnetic actuators more precisely, magnetic encoding strategies have been developed [26–33]. These principles enable 2D soft actuators with defined 3D discrete magnetization profiles to realize sophisticated configurations with programmable shape changes. However, many magnetically encoded structures adopt soft materials, such as elastomer (modulus ≤10 MPa), which lack the property of high-frequency actuation according to the theory of vibration mechanics. As a result, seeking a strategy for realizing morphable 3D mesostructures with high-order deformation and untethered high-frequency actuation (and thus enabling the execution of complex tasks) is still a challenge and an urgent requirement.

Here, we introduce a facile method of building magnetically encoded transferable 3D mesostructure (METM) to achieve high-order deformation and high-frequency actuation. The METM is mechanically guided by a magnetic 2D precursor, which is composed of polyethylene terephthalate (PET) film as a skeleton, and encoded magnetic domains as actuating components. Thanks to the superior shape-retention and adhesion properties of PET after heat treatment, the METMs are capable of 3D structures preserving after being released from the substrate and transferring to other functional surfaces. The encoded magnetic domains on the structure are defined by precisely reorienting the permanent magnetic particles embedded in ultraviolet (UV) curable resin. Under a static magnetic field, the METMs demonstrate high-order 3D configurations because of the arbitrary distribution of magnetic torque assigned to every actuating domain. Furthermore, finite element analysis (FEA) of the 3D magnetic responses enables the design of more sophisticated METMs with high-order shape-morphing ability, such as combining bending and twisting, and hierarchical and multidirectional deformation in one configuration. Based on arrayed high-order morphable METMs, a colour letter display is integrated to realize visual information delivery. More importantly, due to the modest modulus of PET film (∼3 GPa), the METMs have a high resonant frequency (∼55 Hz) and large deformation (∼66.8%) under an alternating magnetic field. On this basis, a variety of functional devices, such as a liquid mixer, sequential flashlight and sliding robot, are manifested by high-frequency responsive METMs.

## RESULTS AND DISCUSSION

### Fabrication of a METM with high-order shape morphing and high-frequency actuation

**Figure 1.**
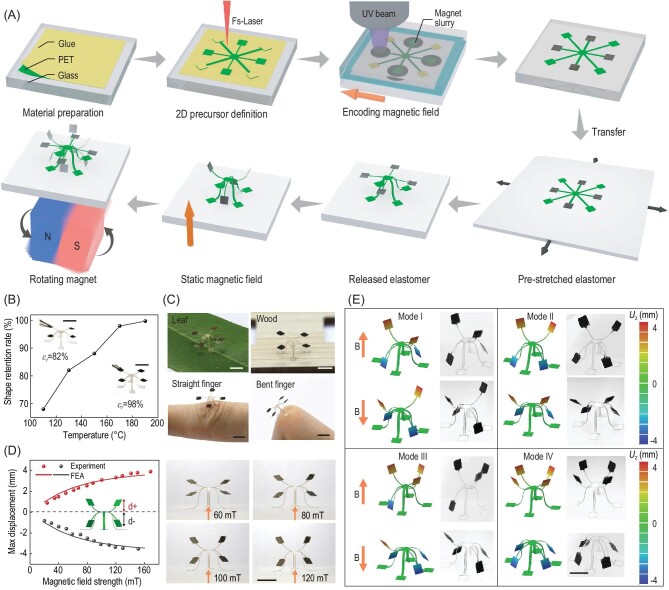
The fabrication process of METMs with magnetically actuated high-order shape morphing and high-frequency actuation. (A) Schematic illustration of the fabrication procedure of a METM and its actuation under static and alternating magnetic fields. (B) The rate of shape preservation of a METM depending on the heat treatment at different temperatures for 10 mins. (C) Four representative functional surfaces bonded with METMs to demonstrate their transferable characteristics. (D) Dependence of the upward and downward experimental displacement of magnetic arms on the magnetic field strength, which is in good agreement with the results predicted by FEA. (E) FEA simulated and experimental 3D configurations of four typical METMs with the same precursor but different discrete magnetization profiles under magnetic field (100 mT). Scale bars: 5 mm.

In this work, the METM is prepared by defining a 2D magnetically coded precursor followed by the mechanically guided assembly, as illustrated in Fig. 1A and Fig. S1, Supplementary Data. The scheme begins with putting PET tape on a glass plate with the glue upwards. The PET tape consists of a bilayer of PET and glue (thickness = 60 μm and <1 μm, respectively). PET is an ideal material for our METM and the subsequent magnetic actuation due to its (i) high mechanical strength (∼3 GPa), with the property of high-frequency response; (ii) large deformation, even under rapid actuation; and (iii) resistance to high temperature (∼200°C) and thermoplasticity, which enables it to maintain its shape after heating. In addition, the glue on the PET tape acts as a facile bonding mode with the pre-stretched elastomeric substrate; this is different to other adhesion types reported before, such as bonding and superglue, which result in difficulty peeling the structure off and which cannot be transferred onto other surfaces, such as skin or leaves. Thus, the glue here introduces a transferable property to the METM. Then, a femtosecond laser is chosen not only to define the 2D precursor but also to remove the glue on the tape (except for at the bonding sites). Additional details about the femtosecond laser processing are shown in Note S1, Movie S1 and Fig. S2, Supplementary Data.

After the definition of the 2D precursor, discrete magnetization profiles are patterned by UV curing of magnetic composite materials. The composite materials are composed of neodymium iron boron (NdFeB) particles and UV resin in a mass ratio of 2 : 1. To obtain permanent magnetic particles, we grind commercial magnets (N35) into microparticles with an average size of ∼7 μm (Fig. S3, Supplementary Data). A mass ratio of 2 : 1 is chosen, considering homogeneous magnetic resin and UV curable thickness, because a ratio of <1 : 1 results in apparent clusters of particles, while a ratio of >2.5 : 1 is difficult to cure. Then, we smear the magnetic resin on the arms of the 2D precursor and follow this with UV curing of discrete magnetization realized by homemade magnetic encoding equipment [30], shown in Figs S4 and S5, Supplementary Data. Before UV light illumination, the hard magnetic particles in the resin are aligned to the direction of the encoded magnetic field, with 50 to 120 mT generated by a 1-inch magnet placed under the fabrication stage (Note S2, Fig. S6 and Movie S1, Supplementary Data).

The permanent magnet is held with the centre fixed, but the azimuthal and polar angles can be independently controlled by two stepper motors, which can generate a 3D magnetic field. After curing, the NdFeB particle orientation is fixed, and the arm can be magnetically actuated. Discrete magnetization profiles are patterned by following the same curing procedure except for the vector of the definition magnetic field when tuning to other arms. The 2D precursor with patterned magnetization is then developed in ethanol for ∼1 minute to remove the uncured magnetic resin. Our system can digitally program magnetic anisotropy with a spatial resolution of ∼50 μm, so a well-defined 2D magnetic coded precursor acquires a magnetization pattern with four discrete domains on its four arms.

The 2D magnetically coded precursor film morphs into the METM via mechanically guided assembly assisted by a homemade electric stretching stage, as shown in Fig. S7 and Movie S2, Supplementary Data. The 2D precursor film is transferred from the above curing platform (glass plate) to a pre-stretched silicone elastomer (${\varepsilon }_{pre} = 90\% $) by facile glue joints. Releasing the pre-strain in the elastomer allows it to return to its original shape, thereby inducing large compressive forces on the 2D precursor film at the bonding sites and bucking it into the corresponding 3D architecture. Upon applying an upward or downward static magnetic field generated by two parallel magnets, the METM in the central zone demonstrates high-order shape morphing (Fig. S8, Supplementary Data). The high-order deformation of the METM and the applications based on this are all actuated by the two parallel magnets in the subsequent sections. When exposed to a high-frequency alternating magnetic field created by a single rotating magnet, the METM shows high-frequency actuation (Fig. S9, Supplementary Data), and subsequent applications based on this are all actuated by the single rotating magnet.

In addition, our system can also fabricate a microscopic METM. In order to increase the processing resolution and reduce the size of the structure, a 10× objective lens is added in the laser processing system (Fig. S10, Supplementary Data) and the diameter of the laser spot decreases from 30 μm to 7 μm. Therefore, a microscopic METM can be fabricated, whose lateral dimension and feature size are 1.1 mm and 20 μm, respectively. The microscopic METM has the same properties as a macroscopic one.

### METMs with transferable properties

Traditional mechanically guided 3D mesostructures rely heavily on pre-stretched elastomeric substrates and return back to their original 2D film when the substrate is removed. Although SMPs [21] and mechanical interlocks [34] have been developed to endow the 3D mesostructures with freestanding ability, the lack of adhesiveness between the structures and surfaces severely restricts their applications. We introduce a PET film with glue to solve this problem. Due to the thermoplasticity of PET material, the constructed METM remains standing after heating above 150°C for 10 minutes and is released from the substrate (Fig. 1B). In addition, the left glue, after laser scanning on the 2D precursor, functions as a facile bonding type for varied surfaces, including leaves, wood, and straight and bent fingers (Fig. 1C). On the one hand, the transferable METMs without substrate can broaden the potential of 3D mesostructures in various circumstances, especially when working on curved or hard-to-bond surfaces (e.g. liquid condition). On the other hand, this adhesion mode enables 3D mesostructures with fast actuation and deformation. To quantify the shape retention of the METMs, we define the distance between two opposite bonding sites of the structure as ${{\rm{d}}}_{\rm{i}}$ and ${{\rm{d}}}_{\rm{e}}$, before and after releasing from the substrate, respectively. Thus, the shape retention ratio ${\varepsilon }_r$ is defined as

(1) \begin{equation*} {\varepsilon }_r = \left( {1 - \frac{{\left| {{d}_i - {d}_e} \right|}}{{{d}_i}}} \right) \times 100{\rm{\% }}. \end{equation*}

It is obvious that ${\varepsilon }_r$ increases with temperature and reaches 97.99% at 170°C and 99.73% at 190°C after baking for 10 minutes, as shown in Fig. 1B. However, some noticeable curling occurs when the temperature is over 200°C. The excellent transferability and adhesiveness of the METMs pave the way for structural adaptability to various surfaces and future applications of magnetic machines.

### High-order shape morphing of diverse METMs

The high-order deformation means that the 3D architectures can morph into a multiconfiguration and have deformation of a high DOF. More specifically, a high DOF applies to bending and twisting, as well as blending and hierarchical and multidirectional deformation in one configuration. By applying a static magnetic field, METMs present some special static magnetic actuation behaviours. Since PET film possesses the modest mechanical strength mentioned above, the hanging magnetic arms on METMs stay nearly horizontal initially, which we choose as the equilibrium state. Upon actuation under an upward or downward magnetic field, the magnetic arms leave the equilibrium position and go up or down, which is shown in the inset of Fig. 1D. The ${d}_ + $ and ${d}_ - $ are defined as the upward displacement and downward displacement, which are positively related to the applied magnetic field strength (Fig. 1D). However, due to the limitation of strength and length of arms, ${d}_ + $ and ${d}_ - $ are difficult to increase while further increasing magnetic field strength above 120 mT when the displacement reaches its maximum of ∼4 mm. The above experimental displacement results are in good agreement with the results predicted by FEA.

**Figure 2.**
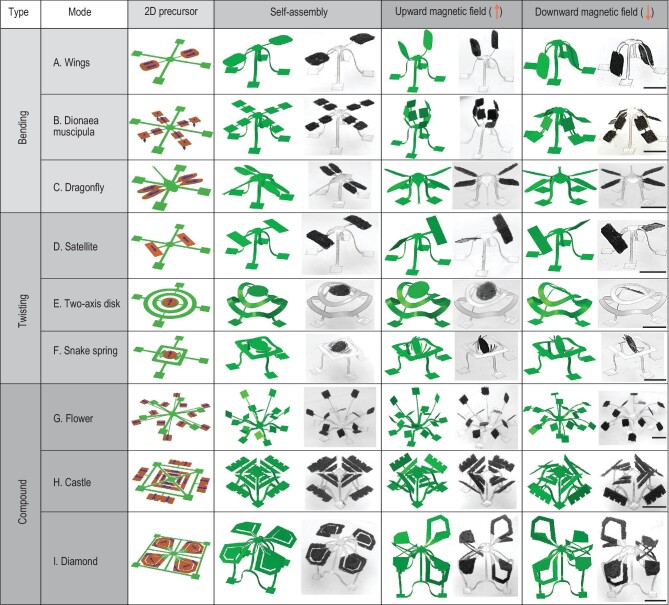
(A–I) Schematic of 2D precursors with magnetization profiles, FEA simulations and optical images of assembled 3D mesostructures, shape morphing of 3D mesostructures under upward magnetic field and downward magnetic field (∼100 mT). (A–C) Denote the bending modes; (D–F) present the twisting modes; (G–I) demonstrate the multi-level and blending modes. Scale bars: 5 mm.

Due to the high design freedom of our 3D magnetization system, discrete magnetic domains can be arbitrarily created to form distinguished magnetization patterns in the same METM. The METM has four arms, and each arm can be magnetized in two opposite directions. Therefore, we can present 2^4^=16 choices of magnetic definition for the same METM. To demonstrate the magnetic programmability and magnetic responsiveness, four typical METMs (Modes I–IV) with the same precursor but different discrete magnetization profiles are fabricated and actuated to multiconfiguration by the upward or downward magnetic field shown in Fig. 1E. To realize desirable 3D shape morphing under a magnetic field, FEA is carried out to design the magnetic anisotropy (Note S3, Supplementary Data). The FEA results agree well with the experimental results, suggesting that the FEA simulation is capable of guiding the design and magnetic actuation of a METM. With the above systematic and computationally guided design approach, nine representative examples of METMs are designed and fabricated based on bending mode (wings, Dionaea muscipula and dragonfly, Fig. 2A–C), twisting mode (satellite, two-axis disk and snake spring, Fig. 2D–F), and multi-level and blending mode (flower, castle and diamond, Fig. 2G–I). These configurations with high-order shape morphing offer the ability to conduct complicated tasks.

### Switchable colour letter display based on high-order morphable METM array

**Figure 3.**
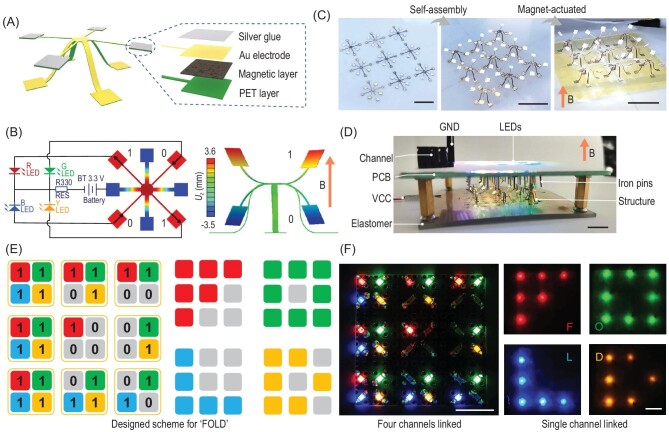
Functional demonstration of the high-order morphable METM array-based switchable colour letter display. (A) Schematic illustration of the conductive METM device and expanded view of the layout. (B) Schematic showing the integration of one circuit unit of a four-channel switchable LED display, and magnetic control deformation of the conductive METM device. (C) The 3 × 3 conductive METM array of 2D precursors, assembled 3D structures and 3D encoded shape morphing. Scale bars: 8 mm (left), 12 mm (middle), 12.5 mm (right). (D) Construction of the LED colour letter display module. Scale bar: 9 mm. (E) A representative designed colour letter display of ‘FOLD’ with four channels connected (all letters displayed) and a single channel connected (one letter displayed). (F) Corresponding experimental colour letter display. Scale bars: 10 mm.

METMs with the character of high-order deformation can be integrated in the circuit to display switchable colour letters by magnetic actuation. To make the METM conductive, multiple functional materials and components are incorporated in the structure via the multi-step microfabrication processes illustrated in Fig. 3A. A layer of gold (30 nm) is first evaporated on the surface so that the structure obtains global conductivity. To further enhance the conductivity of the METM, a layer of conductive silver glue is smeared on the magnetic domains to a thickness of ∼50 μm. An expanded view of the selected area in the right of Fig. 3A shows the layout of the conductive METM device. Because the device possesses four magnetically coded conductive arms, four-channel switchable light-emitting-diode (LED) display circuits are integrated by connecting four arms into four independent circuits with red (R), green (G), yellow (Y) and blue (B) LED lights (Fig. 3B). Upon applying a magnetic field, the encoded arms capable of high-order deformation demonstrate upward movement and are connected to the circuit contact, which denotes on-state with a light on, represented by ‘1’, while downward deformation denotes off-state with the light off, represented by ‘0’.

According to the method introduced above, a 3 × 3 structures array, with the conductive METM device as the modular unit, was designed. The fabrication steps are illustrated in Fig. 3C. A total of 3 × 3 four-arm 2D precursors with a bonding distance of 3 mm are attached to a pre-stretched elastomer and assembled into 3D architectures after stress release, followed by conductive layer coating. The prepared METMs@elastomers are then integrated into circuits to construct the LED colour letter display module (Fig. 3D). Details about the circuit design are shown in Fig. S11, Supplementary Data. The printed circuit board (PCB) has 6 × 6 arrayed iron pins as the module negative terminal, and the corresponding 6 × 6 METM array is the module positive terminal. The LED colour pattern based on the 6 × 6 display board achieves visual information delivery when every METM beneath the board is well encoded and actuated simultaneously under a magnetic field. Figure 3E presents the coloured letters ‘FOLD’, displayed as representative examples in this manner. A four-colour LED light pattern was designed with all four channels connected, and the experimental results are shown in the left of Fig. 3E and F. By choosing the ‘red’ channel while cutting off other channels, only the red letter ‘F’ is displayed. Subsequently, when switching to ‘green’, ‘blue’ and ‘yellow’ channels, green ‘O’, blue ‘L’ and yellow ‘D’ are lit up in turn (Fig. 3F). As the module demonstrated here is based on a 3 × 3 METM array, further efforts to increase array size can be made to exhibit a more sophisticated pattern, which paves the way for constructing multifunctional and responsive devices.

### High-frequency actuation of METM with large deformation

**Figure 4.**
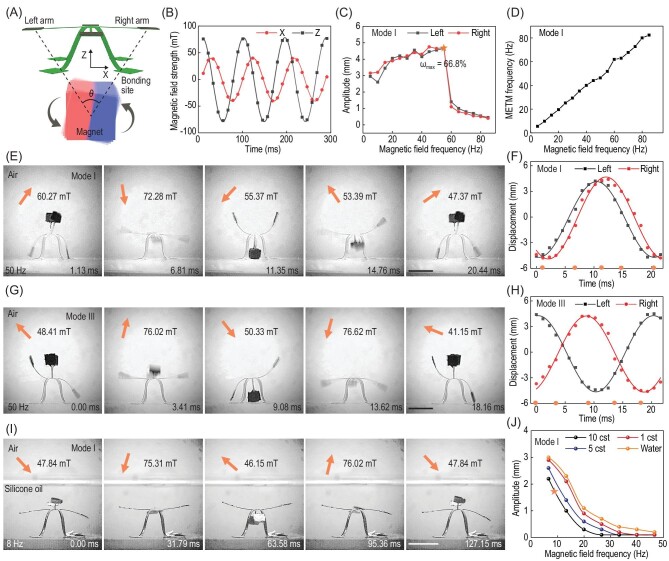
High-frequency actuation of a METM with large deformation under an alternating magnetic field. (A) Schematic diagram of an actuated METM under a high-frequency rotating magnet. (B) Magnetic field strength along the X direction and Z direction in the XOZ flat of the high-frequency alternating magnetic field. (C) Actuation amplitude of the left and right arms depending on the frequency of the alternating magnetic field. (D) Actuation frequency of arms depending on the frequency of the alternating magnetic field. (E and F) Optical time-lapse image of METM deformation (Mode I) actuated by a 50 Hz alternating magnetic field in the air, and its left and right arms’ displacement-time curve. (G and H) Optical time-lapse image of METM deformation (Mode III) actuated by a 50 Hz alternating magnetic field in the air, and its left and right arms’ displacement-time curve. (I) Optical time-lapse image of immersed METM deformation (Mode I) actuated by a 7.8 Hz alternating magnetic field in silicone oil with 10 cst viscosity. (J) Dependence of the immersed METM arms’ displacement on the solution viscosity and magnetic field frequency. Scale bars: 5 mm.

The METM using PET film as a skeleton, with modest modulus (∼3 GPa), and holding magnetically encoded arms with well-designed structure size, can shake its arms with large deformation and show high-frequency vibration characteristics when actuated in an alternating magnetic field. The METM is first actuated at high-frequency in air, and the amplitude of the arms varies with the frequency of the driving magnetic field generated by a rotating magnet, which is located under the structure in the XOZ plane (Fig. 4A). The reason to use a permanent magnet is that it can generate a high strength magnetic field at high frequency without consuming much energy. To investigate the magnetic force applied to the METM, the centre point between four arms is chosen as the reference point, with its magnetic field strengths measured. Detailed statistics about the X component and Z component of magnetic field strength at the centre point are shown in Fig. 4B. We can see two classic sinusoidal curves with the X component 96 degrees phase-leading to the Z component, and the Z amplitude (80 mT) is larger than the X amplitude (40 mT). Then, the responsiveness of METM (Mode I) in the air is presented by studying the amplitude and frequency under an alternating magnetic field. As illustrated in Fig. 4C, the amplitude (A) increases with the frequency of alternating magnetic field and remains unchanged at nearly 4.7 mm (from 45 Hz to 55 Hz, corresponding to resonance frequency range), while suddenly dropping to 1.1 mm (60 Hz). We define a deformation rate to quantitatively evaluate the deformability of a METM:

(2) \begin{equation*} \omega = \frac{A}{L} \times 100\%, \end{equation*}

where L is the length of a single arm. Due to the amplitude enhancement effect of resonance, the maximum deformation rate can reach 66.8%. According to the vibration theory, the vibration of METM in the air is the underdamping state. Figure 4D shows that the vibrational frequency of the arm is highly consistent with the frequency of the alternating magnetic field. Next, Modes I and III are chosen as typical METMs and actuated at 50 Hz in air. Figure 4E demonstrates five captured moments corresponding to five representative configurations of Mode I during vibration, with different magnetic field magnitudes and directions. The displacements of the left and right arms within one vibration period are presented in Fig. 4F. We can see a phase error between the left and right arms mainly caused by the $\theta $ shown in Fig. 4A. Correspondingly, Fig. 4G and H shows five captured moments, and the displacements of two arms of Mode III in vibration, and a 90° phase difference with a phase error can be predicted in Fig. 4H. After the METMs are set in a liquid environment with higher damping coefficient, a noticeable decrease in the arms’ amplitude can be observed in Fig. 4I, which shows that the METM is in an overdamped state. Further research on METM vibrating in different viscous solutions is demonstrated in Fig. 4J. We can conclude that the amplitude of the arms decreases with increasing frequency of the alternating magnetic field and the viscosity of the solution. To the best of our knowledge, the large deformation and high-frequency actuation of morphable 3D mesostructures have never been reported before and can guide the design of the following functional devices.

### Multifunctional applications of high-frequency actuated METMs

**Figure 5.**
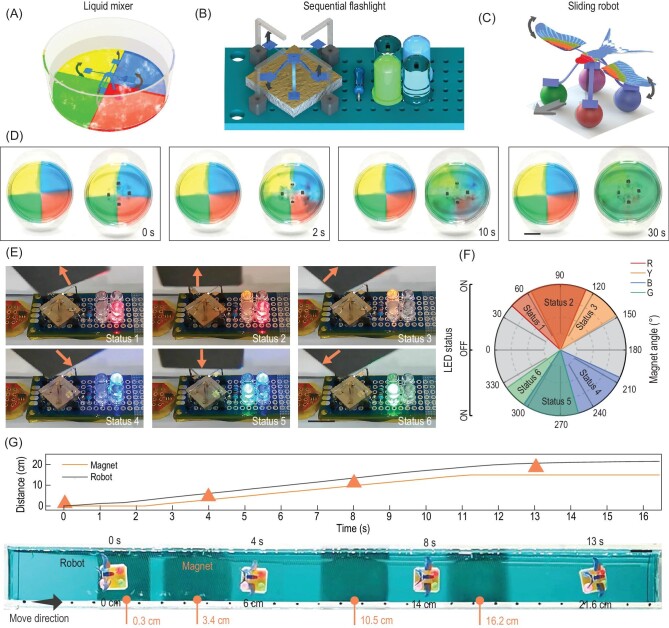
Multifunctional applications of high-frequency actuated METMs. (A) Schematic diagram of the high-frequency liquid mixer. (B) Schematic diagram of the four-colour flashing light. (C) Schematic diagram of the sliding robot on the water surface. (D) Sequential view of the mixing process of four-colour water stirred by a liquid mixer (right) and natural diffusion as a control group (left). (E) Four-colour flashing light with fast switching between six statuses in sequence, with respect to the magnetic field direction (along the arrows). (F) Pie chart of flashing light with six statuses corresponding to the direction of the magnet. (G) Sliding robot gliding on the surface of water actuated by a high-frequency rotating magnet, and the corresponding motion displacement of the flying robot and magnet. Scale bars: 10 mm.

Due to the high-frequency actuation, METMs can have distinct applications in liquid mixers, sequential flashlights and biomimetic sliding robots. First, a high-frequency liquid mixer is constructed by pasting a four-arm METM (Mode I shown in Fig. 1E) in the bottom middle of a petri dish loaded with four-colour equal-area water, as shown in Fig. 5A. Further details are in Fig. S12A and Note S4, Supplementary Data. When exposed to a high-frequency alternating magnetic field, the METM shakes its arms at high frequency and mixes the four-colour water into homogeneous monochromatic liquid within 40 s. The control group takes ∼300 mins to mix uniformly (Fig. 5D and Movie S3, Supplementary Data). To achieve a minimized mixing time, we establish a facile model based on maximized water discharge in unit time (Note S5, Supplementary Data). After calculation, 13 Hz is chosen as the rotational frequency of the magnet corresponding to the maximal water discharge and minimum mixing time (40 s). To quantitatively monitor the degree of liquid mixing, the RGB values of the four-colour area are gathered, as shown in Fig. S13, Supplementary Data. A microscopic liquid mixer is integrated into a microfluidic channel to mix the coloured liquid rapidly (Fig. S14, Supplementary Data). The mixing is realized by the high-frequency actuation of a microscopic METM. After 64 s of mixing, the yellow fluid and blue fluid with a clear boundary transform into uniform green fluid. Details about the mixing process are shown in Movie S4, Supplementary Data.

In the above switchable colour letter display, the METM array with encoding information act as relays to conduct the circuit in static magnetic field. When exposed to an alternating magnetic field in combination with high-frequency actuation, the METM can sequentially conduct the circuit and realize the sequential flashlight. Figure 5B presents a four-colour flashing light as a representative example actuated by a high-frequency alternating magnetic field. The conductive METM device we choose here is Mode I, which is shown in Fig. 1E. As illustrated in Fig. 5E, a cubic permanent magnet is fixed 15 mm above the conductive METM device (Fig. S12B, Supplementary Data). When the magnet rotates, the magnetically encoded arms of the METMs contact the pins in sequence, thus achieving LED lightfast flashing. There are three contact situations between the METMs and pins (no contact, one-arm contact and two-arm contact) corresponding to no light on, one light on and two lights on, respectively, when the magnet rotates a full turn. To quantitatively describe how the status of flashing light varies with magnet rotation, we define a magnet angle α as the angle between the N-pole of the magnet and the horizontal plane. The N-pole pointing to the left stands for α = 0 and α > 0 when the magnet rotates clockwise. As shown in Fig. 5F, when α = 42–63°, the red light stands for status 1. As the magnet turns (α = 63–112°), the yellow light is lit, while the red light remains on (status 2). Finally, only the yellow light is shining (status 3) during α = 112–148°, followed by all lights off. A similar process happens in the next half-circle. Status 4 (blue light on) corresponds to α = 204–254°. Then, status 5 with blue light on and green light on corresponds to α = 254–303°, followed by status 6 with only green light on (α = 303–324°). Additional details are in Fig. S15 and Note S6, Supplementary Data. As the magnet rotates at high speed, a four-colour LED light with six statuses achieves fast flashing (Movie S5, Supplementary Data). By changing the magnetization profiles, many other flashing light-on modes can be designed and realized.

Inspired by water striders sliding on water in nature, a sliding robot is designed and fabricated to achieve the same functionality with different driving forces. Figure 5C shows a schematic illustration of a sliding robot with two magnetically coded wings, which is transferred onto four foam balls on a piece of adhesive tape. The robot floats on the surface of the water in an oblong water channel (30 cm long, 2 cm wide and 1.5 cm deep) with a rotatable magnet beneath (Fig. S12C, Supplementary Data). Initially, the static magnet is below the robot. Once the magnet starts to rotate, the sliding robot flaps its wings at high frequency and glides forward because the wings interact with the surrounding air and generate a forward driving force. In Fig. 5G, the sliding robot generates a straight moving trajectory in the water channel actuated by a high-speed rotating magnet (position shown by the arrow), keeping behind the structure all the time. The relative positions of the robot and magnet during the movement show that the robot (black line) keeps ahead of the magnet (orange line), which indicates that the sliding robot is actuated by aerodynamic force (Movie S6, Supplementary Data). In comparison, if the magnet is moving at the same forward speed without rotating, the magnet keeps ahead, which means the flying strider is dragged forward by magnetic field force rather than aerodynamic force (Movie S6, Supplementary Data). The driving mechanism that accounts for the sliding robot, based on a simplified model, is shown in Figs S16, S17 and Note S7, Supplementary Data.

## CONCLUSION

In summary, the current designed METM, which has a reconfigurable 3D mesostructure, is responsible for high-order shape morphing and high-frequency actuation (Fig. S18, Supplementary Data). By using a homemade magnetic domain defining system, the integration of discrete magnetization profiles onto the 3D mesostructure enables high-order shape morphing. Moreover, reconfigurable 3D mesostructures with favourable moduli contribute to the distinct abilities of high-frequency actuation and large deformation under an alternating magnetic field. Additionally, the high-frequency vibration properties of the METMs in air (underdamping) and in liquid (overdamping) have been systematically studied by using both experimental verification and theoretical simulation. Thanks to their transferable merits, arrayed high-order morphable METMs as conductive components are integrated into circuit devices for four-channel switchable colour letter displays. More significantly, functional demonstrations of high-frequency actuated METMs, including a liquid mixer, sequential flashlight and biomimetic sliding robot, are made that allow for reconfigurable mesostructures, and are applicable in robotics and electronics.

Our proposed strategy for the fabrication of a METM, which uses an fs laser pattern combined with UV selective exposure, dramatically simplifies the manufacturing process of morphable 3D mesostructures compared with conventional methods. For further applications of METMs, such as a high-frequency sensor, wireless micro-pump and micro-driver, there is an urgent need for our METMs to miniaturize and become micro- and nanoscale. Future works will focus on adopting advanced manufacturing technologies involving two-photon polymerization [35,36] or semiconductor technics [37,38] to enable METMs to be applied to the small scale of microsystems (0.1–10 mm). Furthermore, the working distance of current METMs is ∼15 mm, which is enough for the applications of arrayed and fast magnetic actuation demonstrated in this work. The maximum applied magnetic field, generated by a permanent magnet, is 120 mT, which is comparable to that needed in current magnetic actuators [28,31–33]. The miniaturization of METMs will remarkably increase the working distance and decrease the magnetic field, thereby actuating the structures and broadening the applications of METMs in enclosed spaces. To add more functions to the METMs, multiple materials, including metal [39,40] and stimuli-responsive hydrogels [41,42], can be integrated together, which is thought to have the potential to be widely adaptive for microelectronics and biosensing.

## METHODS

### Preparation of materials

NdFeB particles are obtained by grinding commercial N35 magnets that are damaged. They have an average diameter of 7 μm and are mixed sufficiently with UV resin (nanoArch P110, BMF) in a mass ratio of 2 : 1 to form a homogeneous magnetic slurry. The magnetic slurry is placed in a vacuum degassing chamber for 5 minutes to remove the bubbles.

### Sample fabrication

A schematic of the fabrication process can be found in Fig. S1, Supplementary Data. Firstly, we cut a 30 × 70 mm PET type and place it on a glass slide. Then we put it in our femtosecond laser processing system (Chameleon Vision-S, from Coherent Inc., Santa Clara, CA, USA). The femtosecond laser, with a spot diameter of 30 μm and exposure dose of 2.83 × 10^7^ mw/cm^2^, is used to remove most of the glue on the PET type but leave bonding sites reserved. Then we repeat the scan 30 times to cut the shape and paste it to the other glass slide by the bonding sites.

Then we smear the magnetic slurry on the 2D precursor which is covered by a silicone mould with a 25 × 25 × 0.2 mm hole. And then we put a glass slide on the top to limit the slurry thickness. We reverse the sample and set it into our digital light processing (DLP) system.

The 2D precursor is put on a three-axis displacement platform with the magnetic slurry under the objective lens. Then the stepper motors orient the magnet to generate the desired magnetic field, which reorients most of the magnetic particles in the magnetic slurry. Once the motors reach the desired configuration, the motor that connected directly to the magnet performs a damped oscillation that has an initial amplitude of 20°, to bring the particles into alignment faster. After 10 cycles of oscillation, the DLP controller loaded with a predesigned PNG modulates the UV light into desired pattern and the exposure time is set to be 2 s. When we start to pattern, the DLP mirrors array forms a patterned light field to cure the selected region of the magnetic slurry, initiating polymerization and freezing the magnetic particles within the area. The UV laser power is 10.83 mw and the size of UV light spot at the focus is 4.2 mm wide and 7.5 mm long. So, the exposure dose is calculated as ∼34.38 mw/cm^2^. Those steps are repeated optionally until all the regions are patterned and cured properly.

Then we remove the top cover glass slide and silicone mould and put the sample in alcohol to remove the unaggregated magnetic slurry. Then we peel it off from the glass slide and paste it on a pre-stretched elastomer that is stretched by a homemade two-axis stretching platform. We release the elastomer using four motors and the 2D precursor is assembled into a 3D mesostructure guided by mechanical force. Finally, we can put the 3D mesostructure in a designed magnetic field or release it to a fixed 3D mesostructure after heating above 150°C for 10 minutes.

## Supplementary Material

nwac163_Supplemental_Files
